# Kidney Injury Molecule-1 as a Biomarker for Renal Cancer: Current Insights and Future Perspectives—A Narrative Review

**DOI:** 10.3390/ijms26073431

**Published:** 2025-04-06

**Authors:** Dragoș Puia, Marius Ivănuță, Cătălin Pricop

**Affiliations:** 1 “Grigore T Popa”, Faculty of Medicine, University of Medicine and Pharmacy, 700115 Iasi, Romania; dragos-puia@umfiasi.ro (D.P.); catalin.pricop@umfiasi.ro (C.P.); 2 Department of Urology, “Dr. C.I. Parhon” Clinical Hospital, 700503 Iasi, Romania; 3 Center for Morphological and Spectroscopic Analysis of Urinary Stones “Michel Daudon”, 700503 Iasi, Romania

**Keywords:** KIM-1, renal cell carcinoma, biomarker, renal tumour, prognosis

## Abstract

Kidney injury molecule-1 (KIM-1) is a transmembrane protein that is significantly upregulated in renal cells following injury. It has considerable potential as a biomarker for diagnosing and monitoring renal cell carcinoma (RCC). This review examines KIM-1 expression across multiple biological sources—including tissue, blood, and urine—and highlights its strong association with RCC risk. Clinical studies have shown that KIM-1 levels decline within weeks after nephrectomy, underscoring its utility in assessing therapeutic response. Additionally, urinary KIM-1 levels correlate with histopathological outcomes following cisplatin treatment, supporting its role as a non-invasive marker for treatment effectiveness. Despite these promising findings, several challenges remain. These include variability in assay performance and the modulatory effects of the tumour microenvironment on KIM-1 expression. Overcoming these technical limitations is crucial for integrating KIM-1 into clinical workflows. Furthermore, its potential role in guiding combination therapies—such as tyrosine kinase inhibitors (TKIs), immune checkpoint inhibitors (ICIs), and mTOR inhibitors—could enhance therapeutic precision while minimizing toxicity. Continued research is essential to validate these applications and facilitate the routine clinical use of KIM-1 in RCC management.

## 1. Introduction

Identifying Kidney Injury Molecule-1 (KIM-1) marked a key advancement in nephrology by introducing a new renal damage and illness biomarker. KIM-1 can be detected in various biological samples, including urine, blood, and tumour tissue, depending on the clinical context. The HAVCR1 gene encodes the protein, which is distinguished by its unusual structure comprising an immunoglobulin-like domain, a mucin domain, and a transmembrane domain. The architecture of KIM-1 is essential to its functionality. The extracellular portion of KIM-1 has an immunoglobulin-like domain that facilitates cell-cell interactions and ligand binding. This domain is succeeded by a mucin domain, abundant in threonine, serine, and proline residues, which enhances the protein’s elongated shape and its capacity to interact with other molecules. The transmembrane domain secures KIM-1 to the cell membrane, whereas the cytoplasmic tail includes phosphorylation sites essential for intracellular signalling pathways.

KIM-1 has become a crucial biomarker in the diagnosis and prognosis of renal cell carcinoma (RCC). This biomarker’s value resides in its capacity to identify early-stage RCC and track disease development, which is essential for enhancing patient outcomes. KIM-1 is a type I transmembrane glycoprotein that is markedly upregulated in renal tubular cells following injury. Its expression is minimal in normal kidney tissue but significantly elevated in the presence of renal pathology, including RCC. This differential expression makes KIM-1 a valuable biomarker for detecting renal cancer. The efficacy of KIM-1 as a prognostic indicator for RCC decreases with the prolongation of the interval between blood collection and diagnosis, underscoring the need for early identification [1].

The increased incidence of RCC in males, representing approximately two-thirds of worldwide cases and fatalities, might be partially ascribed to a higher prevalence of modifiable risk factors like smoking, hypertension, and obesity [2]. Combining these variables and the reduced survival rate in males highlights the necessity for effective biomarkers such as KIM-1 to enable early diagnosis and management. The implementation of KIM-1 in clinical settings requires further validation. The overlap in KIM-1 concentrations between renal cancer patients and specific controls suggests that, while KIM-1 acts as a useful marker, it may not be conclusive on its own. This necessitates the integration of KIM-1 with additional diagnostic markers or imaging methods to enhance diagnostic accuracy [3].

The reduction in KIM-1 levels following nephrectomy indicates its potential utility in monitoring disease progression and treatment response, which could guide follow-up protocols for RCC patients [4].

Identifying appropriate biomarkers for RCC is critically important due to the consequences of late diagnosis and the lack of effective disease surveillance and methods for predicting drug efficacy [5].

KIM-1, a homolog of Tim-1 in rats and HAVCR-1 in humans and primates, is a promising candidate for addressing these challenges [6]. RCC is characterized by complex pathophysiology involving numerous genetic and molecular alterations. Chromosomal complexity is a critical predictor of RCC development, encompassing systemic spread, aggressiveness, and survival. Chromosome instability leads to genomic lesions that disrupt gene expression patterns, thus accelerating RCC’s metastatic cascade. The histological and ultrastructural criteria for differentiating between benign and malignant renal epithelial tumours are unreliable, except for oncocytomas and small, low-grade papillary adenomas. This lack of definitive criteria highlights the need for molecular and genetic markers in the diagnosis and prognosis of RCC [7].

## 2. Methodology

This narrative review aims to consolidate current knowledge on the clinical significance of KIM-1 in RCC, with a particular focus on its molecular structure, expression profiles across different biological matrices, and emerging roles in diagnosis, prognosis, and therapeutic monitoring. A structured literature search was performed using three primary electronic databases—PubMed, Scopus, and Web of Science—to identify relevant publications appearing between January 2010 and December 2024. The search strategy employed a combination of Medical Subject Headings (MeSH) and free-text terms, including “Kidney Injury Molecule-1”, “KIM-1”, “renal cell carcinoma”, “RCC”, “biomarker”, “diagnosis”, “prognosis”, “treatment monitoring”, “urinary biomarkers”, and “liquid biopsy”. Boolean operators (AND, OR) were used to maximize both sensitivity and specificity in retrieving pertinent studies.

Eligible studies were required to meet the following conditions: (i) publication in peer-reviewed journals and written in English; (ii) investigation of human subjects diagnosed with renal cell carcinoma, including both clear-cell and non-clear-cell histological subtypes; and (iii) employment of scientifically robust designs, such as randomized controlled trials, prospective or retrospective cohort studies, case–control studies, systematic reviews, or meta-analyses. No restrictions were applied regarding the type of biological sample used to measure KIM-1.

Studies were excluded if they met any of the following criteria: (i) preclinical, in vitro, or animal-based research; (ii) lack of explicit focus on KIM-1 within the context of RCC; (iii) non-peer-reviewed formats, including conference abstracts, editorials, or opinion pieces; or (iv) absence of full-text availability. This methodological approach was devised to ensure a high level of scientific rigour and to facilitate a comprehensive and clinically meaningful synthesis of the literature on KIM-1 as a biomarker in RCC.

## 3. Prognostic Marker

Table 1 provides a structured summary of the most relevant studies exploring the role of KIM-1 as a biomarker in RCC.

KIM-1’s role in kidney injury is multifaceted. It functions as a receptor for phosphatidylserine, a marker of apoptotic cells, facilitating the clearance of these cells through phagocytosis. This role is critical in renal injury, where the removal of apoptotic cells can mitigate inflammation and promote tissue repair [4,8]. Studies have demonstrated that urinary KIM-1 levels are significantly higher in patients with RCC compared to healthy controls, indicating its potential utility in non-invasive cancer diagnostics [1,4].

One of the main advantages of KIM-1 is its detectability in urine; however, although urine sampling is convenient and non-invasive, the kinetics of KIM-1 excretion may vary depending on renal function. In well-functioning kidneys, KIM-1 may remain in circulation longer before being filtered, potentially delaying its appearance in urine. Additionally, urinary KIM-1 levels can rise in patients with early kidney injury unrelated to cancer, which may limit its specificity as a cancer biomarker [1,6].

Early-stage RCC presents a critical intervention window where identifying reliable biomarkers can significantly impact patient outcomes. Studies have shown that elevated levels of KIM-1 in plasma or urine can indicate the presence of RCC even before clinical diagnosis. According to Scelo et al., KIM-1 levels can be measured in prediagnostic samples, allowing for the potential early detection of RCC [1]. According to the authors, RCC’s incidence rate ratio (IRR) was 1.71 for every doubling of plasma KIM-1 concentration, demonstrating a significant connection between higher KIM-1 levels and increased risk.

KIM-1 is predominantly expressed in clear cell renal cancer, the most common subtype of RCC. According to Karmakova and Han et al., KIM-1 expression is significantly higher in clear cell renal cancer tissues compared to other renal tumours, such as chromophobe RCC and benign oncocytomas, where its expression is rare [6,8]. This differential expression underscores the specificity of KIM-1 as a biomarker for clear-cell renal cancer. The ectodomain of KIM-1 is cleaved, allowing it to be identified in urine and blood. Elevated levels of KIM-1 in fluids have been linked to RCC, especially after diagnosis. KIM-1 is a non-invasive biomarker for early-stage RCC that may be evaluated using urine or blood testing. This allows for early diagnosis and monitoring of the illness. Furthermore, the correlation between KIM-1 levels and tumour characteristics, such as grade and size, has been established. Higher KIM-1 expression is associated with more aggressive tumour features, indicating its role in predicting disease progression [9,10]. This association is crucial for stratifying patients based on risk and tailoring treatment strategies accordingly.

KIM-1 may be a valuable tool for differential diagnosis in patients with urothelial tumours with renal parenchymal invasion. According to Białek et al., urinary KIM-1 levels can help differentiate between upper tract urothelial carcinoma (UTUC) and RCC. The median concentration of urinary KIM-1 was lower in RCC patients than in those with urothelial carcinoma (1.35 vs. 1.86 ng/mg creatinine, *p* = 0.04). The study found that while urinary KIM-1 can potentially identify urothelial carcinoma, the sensitivity (33.3%) and specificity (96.7%) at the cut-off value of 3.226 ng/mg creatinine indicate that caution is needed in clinical decision-making [11]

Beyond diagnosis, KIM-1 appears to have prognostic value. Elevated levels have been linked to poor survival outcomes, yet paradoxically, TCGA data show that higher HAVCR1 (KIM-1) mRNA expression in tumours is associated with improved overall survival [12]. Lee et al. proposed a mechanistic explanation: KIM-1 downregulates the pro-metastatic gene Rab27b, leading to fewer lung metastases in experimental models, even in immune-competent settings [12]. This suggests a context-dependent dual role, where KIM-1 may promote immune regulation and inhibit metastasis, despite being a marker of advanced disease [12]. 

Assessing KIM-1 concentrations in urine offers a non-invasive approach to monitoring kidney cancer, rendering it a valuable instrument for clinical use [13,14]. The specificity and sensitivity of KIM-1 as a biomarker have undergone substantial investigation. Research indicates that although KIM-1 has considerable specificity, its sensitivity may fluctuate. In RCC urine samples, the sensitivity of KIM-1 has been seen to surpass 90% in some instances. This can be affected by variables, including sample size and detection methodology. The variability in sensitivity highlights the necessity for further broad and thorough investigations to confirm these findings and improve the reliability of KIM-1 as a predictive instrument.

One of the key advantages of using KIM-1 as a prognostic marker is its ability to reflect the tumour’s biological behaviour. According to Zhang et al., the amount of KIM-1 present in the urine can be influenced by several factors, including the productivity of KIM-1 by individual tumour cells, tumour size, and the pathways through which KIM-1 is excreted into the urine [4]. This makes KIM-1 a dynamic marker that can provide real-time insights into the tumour’s status and progression. In a cohort of 40 kidney cancer patients and 40 healthy subjects, Mijugkovic et al. evaluated Kim-1 and aquaporin-1 (AQP-1) as potential early urinary biomarkers of clear renal cell carcinoma. The authors highlighted that preoperative urinary KIM-1 levels were significantly higher in RCC patients (0.724 ng/mg urinary creatinine) than in controls (0.210 ng/mg). In contrast, preoperative urinary AQP-1 levels were significantly lower in RCC patients (0.111 ng/mg) compared to controls (0.202 ng/mg). Furthermore, there was a positive correlation between preoperative uKIM-1 levels and tumour size, grade, and stage, indicating that higher levels were associated with more advanced disease [10]. 

Integrating KIM-1 into clinical practice also offers the potential for continuously monitoring disease progression. The non-invasive nature of urine sampling allows for repeated measurements over time, providing a longitudinal view of the patient’s condition. This can be particularly useful in detecting early signs of recurrence or progression, enabling timely interventions [14,15]. Despite its potential, there are limitations to using KIM-1 as a prognostic marker. Therefore, further research is needed to address these limitations and establish standardised KIM-1 measurement and interpretation protocols.

**Table 1:** Overview of key studies on KIM-1 as a biomarker in RCC.

Authors	Study Type	Population	Methodology	Key Findings	Conclusions
Shalabi et al. (2013) [9]	Clinical Study	46 RCC patients	Urinary KIM-1 and NGAL via ELISA	KIM-1 correlates with tumor grade	KIM-1 is a useful diagnostic marker for RCC, showing strong correlation with tumor grade
Morrissey et al. (2011) [3]	Clinical Study	67 RCC patients, 55 controls	Urinary KIM-1 and NGAL	KIM-1 correlates with tumor size and post-nephrectomy reduction	KIM-1 is a highly specific and non-invasive RCC biomarker, useful for early diagnosis and monitoring
Scelo et al. (2018) [1]	Epidemiological Study	190 RCC patients	Plasma KIM-1 via ELISA	High KIM-1 predicts poor prognosis	Plasma KIM-1 may serve as an early detection biomarker, predicting poor prognosis and recurrence risk
Mijugkovic et al. (2017) [10]	Prospective Follow-Up	40 RCC patients	ELISA for urinary KIM-1	KIM-1 correlates with tumor size and grade	Urinary KIM-1 is a reliable, non-invasive tool for diagnosing and monitoring RCC
Zhang et al. (2019) [4]	Review	Literature Review	Meta-analysis	KIM-1 is a diagnostic and prognostic marker	KIM-1 serves as both a diagnostic and prognostic biomarker in renal disease and cancer
Lee et al. (2021) [12]	Experimental Study	Cell lines and mouse models	Genetic manipulation of RCC cells	KIM-1 inhibits metastasis and is linked to survival	Experimental models show KIM-1 inhibits metastasis and may improve survival outcomes in RCC patients
Karmakova et al. (2021) [6]	Review	Literature Review	Systematic review	KIM-1 functions in renal injury and cancer progression	Review highlights KIM-1’s role in renal pathology, from kidney injury to cancer progression
Han et al. (2005) [8]	Histological Analysis	40 RCC patients	Immunohistochemistry	KIM-1 is highly expressed in RCC tissue	KIM-1 is an RCC-specific marker in tissue samples, supporting its role as a urinary biomarker
Anandkumar DG et al. (2023) [16]	Prospective Study	180 RCC patients	Prognostic Value of KIM-1 in Renal Cancer	KIM-1 expression analysis	KIM-1 is a reliable marker for RCC aggressiveness and treatment response prediction

## 4. Comparison with Other Biomarkers

Table 2 presents a comparative analysis of KIM-1 and other biochemical markers commonly evaluated in renal tumours, highlighting their sample type, diagnostic relevance, advantages, and limitations.

KIM-1 has emerged as a significant biomarker in renal cancer diagnostics, particularly compared to other biomarkers, which require invasive tissue sampling. KIM-1’s presence in urine and plasma makes it particularly attractive for repeated, real-time clinical monitoring [7].

To better understand KIM-1’s potential utility, it is essential to distinguish its roles across key clinical domains: screening, diagnosis, monitoring, and prognosis. KIM-1’s primary advantage compared to other biomarkers is its detectability in urine, providing a non-invasive option for patients. This is especially advantageous in contrast to tissue-based biomarkers, which frequently necessitate invasive procedures like biopsies. Tissue KIM-1 levels are elevated in patients undergoing radical nephrectomy compared to those undergoing partial nephrectomy, suggesting its effectiveness in identifying clear cell RCC [16]. Sensitivity is essential for the early detection and monitoring of disease progression.

However, KIM-1 is not without limitations. One significant challenge is its presence in conditions other than renal cancer, such as acute kidney injury. This can potentially lead to false positives if KIM-1 is used as a stand-alone screening tool. Nonetheless, most patients with localised RCC present with normal renal function, which aids in distinguishing them from other renal diseases. C-reactive protein (CRP) although commonly used as an inflammatory biomarker, lacks specificity for renal malignancies. Its elevation may be confounded by systemic inflammation or infection. However, CRP has demonstrated prognostic relevance, with studies showing correlations between elevated CRP levels and poorer survival outcomes in RCC patients [17,18]. Still, it is not suitable for diagnostic purposes alone [17]. According to Chen et al., CRP levels correlate with recurrence-free survival (RFS) and overall survival (OS) in non-metastatic clear cell RCC, with optimal cut-off values varying by race. The highest mean level was observed in Afro-Americans (5.00 mg/L), while the lowest was in Asians (0.9 mg/L) [18]. Moreover, according to Hirata et al., the C-reactive protein–albumin–lymphocyte (CALLY) index has emerged as a robust predictor of postoperative recurrence, outperforming traditional factors such as Fuhrman grade. The authors identified three key predictive factors for progression-free survival (PFS) following surgery: the presence of sarcomatoid features, a Fuhrman grade of 4, and a low C-reactive protein level [19]. Other biomarkers, such as NGAL (Neutrophil Gelatinase-Associated Lipocalin), have been investigated for their involvement in renal cancer. NGAL, akin to KIM-1, was discovered via differential gene expression studies and has demonstrated upregulation subsequent to acute kidney injury [3]. Nevertheless, the clinical applicability of NGAL in renal cancer is less investigated compared to KIM-1. Morrissey et al. reported that the sensitivity and specificity of NGAL in identifying kidney cancer were limited, with an area under the curve (AUC) of 0.64, suggesting it is not a reliable biomarker for this condition. KIM-1 strongly correlates with tumour size (Spearman’s r = 0.66) and demonstrates solid diagnostic accuracy (AUC = 0.75), supporting its value as a promising biomarker for renal cancer [3].

Furthermore, integrating KIM-1 with other biomarkers could enhance diagnostic accuracy. For instance, combining KIM-1 with markers of systemic inflammation, such as TNF receptors, has shown a strong association with RCC risk, indicating that a multi-marker approach could improve early detection and risk stratification [1]. This strategy aligns with the need for biomarkers to represent renal function while minimising collinearity comprehensively. A study by Peng et al., conducted on 176 healthy participants, revealed a positive correlation between urinary KIM-1 levels and age, suggesting that KIM-1 may reflect kidney ageing processes. The study developed a predictive model for kidney age using KIM-1 alongside other biomarkers, achieving a significantly adjusted R^2^ of 69.5% [13].

KIM-1 also offers value in treatment monitoring, as its levels reflect changes in tumour activity and therapeutic response over time. The role of KIM-1 in treatment response prediction remains under investigation. Pre-clinical studies indicate that KIM-1 could serve as a predictive biomarker for adjuvant immune checkpoint inhibition, especially in cases of vascular supply limitations in macroscopic tumours. The predictive capability of KIM-1 may establish it as an important instrument for diagnosis and informing therapeutic choices. The prognostic performance of KIM-1 has been assessed in comprehensive studies, indicating its effectiveness in evaluating the risk of recurrence following nephrectomy in RCC. Plasma KIM-1, due to its minimally invasive nature, enhances risk stratification, which is essential for post-surgical patient management. Xu et al. report that KIM-1 levels decline following effective treatments such as nivolumab, which is associated with enhanced progression-free survival [20]. The prognostic value of KIM-1 differentiates it from other biomarkers that may lack comparable predictive insight. Comparison with other biomarkers underscores its distinct advantages and identifies areas for further investigation, especially in the integration with various markers to improve diagnostic and prognostic precision. Assessing renal function in patients with an acquired solitary kidney following nephrectomy presents notable challenges, with outcomes affected by multiple surgical and physiological factors. Comprehending these factors is essential for enhancing patient care and reducing the risks associated with the progression of chronic kidney disease (CKD). Patients with acquired single kidneys experience elevated functional demands on the remaining kidney, resulting in an increased risk of CKD progression [21]. The potential of KIM-1 as a therapeutic target in renal cancer represents a significant area of interest. Increased KIM-1 expression in tumour tissues, especially in instances of significant lymphovascular invasion, indicates its involvement in tumour aggressiveness and progression. The correlation is additionally substantiated by the finding that patients who undergo radical nephrectomy demonstrate elevated KIM-1 expression compared to those who have partial nephrectomy [10]. This suggests that KIM-1 may serve as a marker for more advanced disease, positioning it as a potential target for therapeutic intervention. In CKD, elevated levels of KIM-1 in the bloodstream are associated with a diminished glomerular filtration rate, underscoring its significance as an early indicator of renal failure progression. This indicates that targeting KIM-1 may slow or prevent the progression of renal impairment in CKD patients, which could also be relevant to its role in renal cancer. The swift emergence of KIM-1 in the urine of individuals with type 1 diabetes during the early phases of diabetic kidney disease highlights its potential as an early marker of renal impairment [22]. The early detection capability may be utilized in renal cancer to identify and treat the disease at an initial stage, potentially enhancing patient outcomes. The increased excretion of KIM-1 in urine is a specific indicator of kidney injury due to its large molecular mass, which inhibits filtration through the glomerular barrier from extrarenal sources [6].

The specificity of KIM-1 positions it as a promising target for therapies designed to address kidney damage at the injury site, which is particularly advantageous in renal cancer where localized treatment is essential. Using liquid biopsy methodologies has enhanced the applicability of KIM-1 in clinical settings. Liquid biopsies, encompassing the examination of circulating tumour cells (CTCs), cell-free tumour DNA (ctDNA), and other tumour-derived metabolites, offer a non-invasive approach for the ongoing assessment of disease progression and therapeutic effectiveness. This method is especially beneficial in kidney cancer, as conventional tissue samples are invasive and may fail to represent the tumour’s heterogeneity [5]. Advanced metabolomic and proteomic analysis have facilitated the development of new biomarkers and the enhancement of established ones such as KIM-1. Semi-quantitative analysis of volatile organic compounds (VOCs) via MetaboAnalyst 5.0 has distinctly differentiated renal cancer patients from healthy individuals, underscoring the potential of combining metabolomic data with KIM-1 measurements for a more thorough diagnostic strategy [23]. Investigating microRNAs (miRs) as urine biomarkers for kidney cancer has created new possibilities for non-invasive diagnostics. MicroRNAs (miRs), which modulate gene expression by binding to the 3′ untranslated region (UTR) of target mRNAs, have demonstrated potential in identifying patients at heightened risk of kidney cancer development. Integrating miR analysis with KIM-1 detection may improve renal cancer diagnosis’s precision and predictive capability [14].

One promising direction is the combination of KIM-1 with other molecular markers such as PDL1 and CXCR4, which have shown prognostic associations in renal cancer. Although these markers have not yet produced more than level III evidence, their integration with KIM-1 could potentially enhance the predictive power for disease progression and patient prognosis [7]. Additionally, epigenetic modifications, such as miRNA and gene methylations, could provide a more comprehensive understanding of tumour biology and its response to treatment. Another area of interest is integrating KIM-1 with immune checkpoint markers like PD-1, PD-L1, and BTN3A1. These markers have been shown to predict metastasis and correlate with the number and location of metastases in renal cancer patients [7]. By combining these markers with KIM-1, clinicians could develop a more robust framework for predicting disease recurrence and metastasis, enabling personalised treatment strategies. Furthermore, whole-exome and genome sequencing, along with transcriptome and epigenome analyses, can provide valuable insights into renal cancer’s genetic and molecular landscape. These techniques, when combined with KIM-1, could assist in identifying specific genetic alterations and pathways involved in the development and progression of tumours. Although still in its infancy, implementing circulating cell-free RNA (cfRNA) analysis holds promise for non-invasive monitoring of tumour dynamics and treatment response. Another integrative approach involves liquid biopsy markers, such as ctDNA and circulating tumour cells (CTCs). These markers can provide real-time information on tumour burden and genetic alterations, crucial for monitoring disease progression and treatment efficacy. Combining KIM-1 with ctDNA and CTCs could enhance the sensitivity and specificity of renal cancer diagnostics, leading to better clinical decision-making and patient management.

## 5. Limitations

Serum KIM-1 has demonstrated potential as a biomarker for the diagnosis and prognosis of renal cancer; however, several limitations warrant consideration. A notable limitation is the variability in KIM-1 concentrations across individuals, complicating the establishment of a universal threshold for clinical application. The concentration of KIM-1 in plasma can vary significantly; some studies indicate that a concentration of approximately 200 pg/mL is associated with a 63-fold increased risk compared to undetectable levels. The variability requires precise calibration and validation of KIM-1 levels across different populations to guarantee accurate risk assessment. A further limitation is the potential impact of other renal conditions on KIM-1 levels. Increased KIM-1 levels are not limited to renal cancer. They can also be observed in various types of kidney injury, including ischemic or toxic damage to the renal proximal tubule epithelium [6]. The overlap may result in false positives, as elevated KIM-1 levels could be incorrectly interpreted as signs of renal cancer when they are attributable to other renal pathologies. The timing of the KIM-1 measurement also poses a challenge. The optimal cutoff thresholds for KIM-1 levels can vary depending on the timing of the measurement relative to disease progression and treatment interventions [15]. For example, KIM-1 levels have been shown to drop significantly after nephrectomy, indicating that the timing of sample collection is crucial for accurate interpretation [1]. The variability of KIM-1 levels can be attributed to multiple factors, including interindividual genetic differences—such as the expression of distinct splice variants (KIM-1a and KIM-1b) with potentially divergent biological behaviour— the influence of comorbid conditions like diabetes, hypertension, chronic kidney disease, and baseline renal function, which may confound interpretation in non-oncologic settings. Moreover, differences in proteolytic shedding dynamics, variations in sample handling and storage, and the glycosylation status of the protein, which can alter epitope accessibility in immunoassays, all contribute to inconsistencies across clinical studies [1,24,25]. Addressing these limitations through standardized methodologies, larger validation cohorts, and improved biomarker integration strategies will be essential to ensure the reliable clinical application of KIM-1 in RCC.

## 6. Conclusions

KIM-1 has demonstrated consistent potential as a biomarker for the early detection and post-treatment monitoring of renal cell carcinoma. Its ability to distinguish malignant from benign renal conditions, along with its elevated expression in RCC tissues and detectable levels in urine and plasma, supports its clinical relevance. In addition to its diagnostic capabilities, KIM-1 may serve as an indicator of therapeutic response and disease recurrence. However, variability in expression due to renal function and comorbidities remains a challenge, underscoring the need for further standardization across populations and platforms. Particular attention should be given to the investigation of KIM-1 in papillary RCC, where its expression profile may differ significantly from clear cell RCC. Understanding these differences could lead to more tailored approaches in biomarker-guided diagnostics and therapy. Continued translational research is needed to refine the clinical application of KIM-1 and establish its integration into multi-marker strategies for RCC management.

## Figures and Tables

**Table 2:** Comparative overview of KIM-1 and other biomarkers used in RCC.

Biomarker	Type of Sample	Characteristics	Limitations	Clinical Utility
KIM-1	Urine, Blood	Non-invasive, effective in early diagnosis and monitoring RCC progression, correlates with tumor size and aggressiveness.	Variability in sensitivity, overlaps with other kidney injuries, standardization issues.	Used for prognosis, treatment response monitoring, and early detection of RCC.
NGAL	Urine	Indicates acute kidney injury, non-invasive detection. Lower sensitivity and specificity for RCC compared to KIM-1.	Limited clinical applicability specifically for RCC diagnostics.	May be useful as a complementary biomarker for kidney function assessment.
CRP	Blood	Reflects general inflammation, correlated with survival outcomes in RCC patients.	Non-specific marker of inflammation, not specific to renal cancer.	Helps assess overall inflammatory status in RCC patients, sometimes used for risk stratification.
CALLY Index	Blood-based	Strong predictor of postoperative recurrence, superior to traditional measures like Fuhrman grade.	General inflammatory marker, limited specificity for RCC.	Can support risk stratification in RCC patients undergoing surgery.
TNFR	Blood-based	Improves RCC risk prediction when combined with KIM-1, strengthens multi-marker diagnostic approaches.	Ineffective as a standalone marker for RCC.	Used in combination with other markers for better diagnostic accuracy.

NGAL—Neutrophil Gelatinase-Associated Lipocalin; CALLY—C-reactive protein–albumin–lymphocyte index; TNFR—Tumour Necrosis Factor Receptors.

## Data Availability

Not applicable.

## Abbreviations

The following abbreviations are used in this manuscript:

KIM-1	Kidney Injury Molecule-1
RCC	renal cell carcinoma
TCGA	The Cancer Genome Atlas
NGAL	Neutrophil Gelatinase-Associated Lipocalin
CRP	C-reactive protein
CALLY	C-reactive protein–albumin–lymphocyte
CKD	chronic kidney disease
DKD	diabetic kidney disease
CTCs	circulating tumour cells
ctDNA	cell-free tumour DNA
VOCs	volatile organic compounds
TNFR	Tumour Necrosis Factor Receptors
